# Identity-Driven Targeted Violence in a College Setting: An Overview of Prevalence and Behavioral Responses

**DOI:** 10.3390/ijerph21101312

**Published:** 2024-09-30

**Authors:** Patrick T. McGonigal, Mario J. Scalora

**Affiliations:** 1 Department of Psychology, University of Nebraska Lincoln, 1220 T St., Lincoln, NE 68588, USA; mscalora1@unl.edu; 2 Patton State Hospital, 3102 Highland Ave., Patton, CA 92369, USA

**Keywords:** harassment, bullying, bias, violence, cyberbullying

## Abstract

Background: Online and offline identity-driven harassment disproportionately affects minoritized college students, contributing to poorer academic performance and attrition. Because victims are often hesitant to formally report incidents, additional research is needed to understand the genuine prevalence of these experiences as well as the responses victims engage in following the incidents. Methods: A large undergraduate sample (*N* = 2000) from a Midwestern university responded to an anonymous survey assessing the frequency of identity-driven behavior occurring on-campus and beyond, in addition to how they responded to harassment. Results: The results unveiled that perpetrators most often targeted an individual’s sex and gender, followed by their sexual orientation and race. Specific behaviors ranged from more frequent, mild forms of harassment (i.e., verbal harassment, invading space) to less frequent, severe forms of harassment (i.e., physical and sexual assault). Victims reported engaging in informal activities following harassment, such as relying on social support or ignoring the perpetrator. Conclusions: The current study unveiled patterns of identity-driven behaviors experienced by college students as well as how they respond to victimization. Future directions and practical implications are discussed.

## 1. Introduction

Incidents of bias and identity-driven harassment comprise a unique form of targeted violence in which perpetrators target victims based on characteristics of their identity (e.g., race, sexual orientation, gender identity, religious affiliation). Identity-driven harassment experiences can range from comparatively mild (e.g., insensitive jokes, slurs) to severe (e.g., physical assault, sexual assault); the core distinguishing factor between this form of harassment and generalized harassment is the identity-driven nature of offenses. Victimization experiences can encompass multiple forms of harassment, ranging from receiving racial epithets to instances of physical following and assault. Research demonstrates the disproportionate amount of targeted harassment and violence rooted in the prejudice, hatred, and bigotry experienced by historically marginalized and minoritized communities, as identity-driven violence is often motivated by prejudice (e.g., racism, homophobia, transphobia) [1,2,3,4,5]. These acts can contribute to severe psychological distress among victims, such as depression, anxiety, and in severe cases, suicidality [6].

A large base of empirical studies suggests that several psychological factors influence an individual’s propensity towards engaging in identity-driven behavior (i.e., prejudicial attitudes, right-wing authoritarianism, social dominance) [7,8]. These psychological variables interact with one’s social context and the broader political atmosphere and heighten the risk of bias perpetration. Given the sensitive nature of incidents of harassment motivated by identity, its genuine prevalence is difficult to ascertain. Nevertheless, the available data suggest that identity-driven harassment is increasing over time; for example, reported hate crimes against transgender and gender non-conforming individuals increased by 84% between 2013 and 2019 [9]. In addition, anti-Asian hate crimes rose significantly in the U.S. during the height of the COVID-19 pandemic [10]. However, the genuine prevalence of bias-motivated harassment and violence is largely unknown, given victims’ hesitancy to report these incidents to formal authorities [11,12]. Underreporting may occur as victims express complex emotions following bias victimization, such as guilt, shame, fear, and distrust of others, that hamper their likelihood of reporting to traditional agencies [11,12]. In an empirical study, Vergani and Navarro found that participants who minimize hate crime seriousness and exhibit less awareness of appropriate reporting channels are significantly less likely to report “less serious” bias incidents (e.g., verbal assaults) [12]. On the other hand, participants were less likely to report grave bias incidents (e.g., physical assaults) if they feared consequences or retribution, had limited access to reporting outlets, or distrusted law enforcement [12].

### 1.1. Identity-Driven Harassment on College Campuses

The university setting draws together students from varying demographic and cultural backgrounds to live and learn alongside one another. University settings inherently pose a heightened risk for crime and targeted violence [13,14]. Firstly, the physical structure of college campuses allows for easier access to facilities, residence halls, and students. Outsiders without university affiliation are typically permitted to walk campus grounds as freely as students, which may raise the risk of harassment of students by community members. Secondly, students and staff within universities operate on regular schedules that dictate their travel, allowing for more opportunities for perpetrators to monitor and surveil the everyday whereabouts of targets. Lastly, students often balance mounting academic and social obligations and navigate complex interpersonal issues such as romantic and sexual relationships, friendships, and peer substance use; thus, accumulating psychosocial stressors may supplement the existing risk of violence on college campuses [13,15].

Unfortunately, identity-driven harassment experiences are not unusual for minoritized students on college campuses [16]. Existing evidence indicates wide-ranging estimates of bias experiences for minoritized students, with at least 32.5% of students of color endorsing being victims of on-campus harassment and at least 36% of undergraduate sexual and gender minorities (SGMs) reporting identity-driven harassment over the last year [17,18]. In a multi-site study, Reason and Rankin found that victimization most often targeted the individual’s gender identity, race, ethnicity, and religious beliefs [19]. These incidents could include vandalization of property with epithets or slurs, physical following of minoritized students to and from class or social activities, physical assault, or intrusive electronic behaviors. A higher proportion of sexual- and gender-minority students (up to 71%) report feeling afraid that they will experience bias during their undergraduate career and tend to express pessimism that administrative authorities will adequately address their concerns [5,17]. Moreover, historically marginalized students are at an increased risk of experiencing recurrent victimization. Among college students, Kammer-Kerwick and colleagues found that SGMs were 74% more likely to experience repeated sexual violence on campus compared to cisgender heterosexual male students in a large multi-site study [20]. Using an Ecological Momentary Assessment (EMA) design, Ortega-Williams and colleagues found that Black youth report pervasive and recurrent instances of racism in public settings (i.e., derogatory remarks) [21]. As victims of identity-driven harassment indicate repeated instances of harassment, the phenomenon is likely more pervasive in nature than isolated.

### 1.2. Cyber-Related Bias and Harassment

As social media and technology continue to shape students’ college experience, cyber-related harassment victimization among college students has similarly risen to the attention of scholars, who describe bias incidents on online platforms as the “new frontier in hate crime offending” [22] (p. 481) [23]. Approximately 20% of college students report victimization via cyberharassment, which disproportionately affects younger students, sexual- and gender-minority students, racial-minority students, and international students [24]. Electronic forms of bias can include sending insensitive texts or emails, making derogatory posts on social media, articulating threats, or the surveillance and monitoring of another person’s online activity, among other problematic behaviors. Technology use has become a mainstream expectation for undergraduate students in the last two decades [25,26], leaving the door open for increased risk of harassment on electronic platforms. Individuals who engage in online forms of harassment tend to exhibit superior online proficiency and awareness and spend more time in cyberspaces [27]. These incidents have damaging implications for victims, resulting in markedly poorer self-esteem, negative affectivity such as anger and depression, and stress [24,28]. In a sample of over 800 college students, Schenk and colleagues found that victims of cyber harassment were significantly more likely to report symptoms of internalizing forms of psychopathology such as depression, suicidality, anxiety, and paranoia [29].

### 1.3. Impact of Identity-Driven Harassment

Offline and online harassment compounds the multitude of additional psychosocial stressors inherent to the college experience (e.g., academics, establishing social connections) and affects the victimized individual, their loved ones, and the campus climate more broadly. College students who endure victimization are more likely to receive poorer grades in class, discontinue their education, and experience disruptions and strain in their relationships, and are less likely to remain active in recreational and social activities on campus [23,28,30]. For example, Mengo and Black collected retrospective academic data from 145 victimized undergraduate students who took part in an on-campus violence prevention program following victimization. The researchers found significant reductions in Grade Point Average (GPA) after self-reported physical and sexual violence victimization [30]. On-campus victimizations may also impact social support, as family members and friends connected to the victim may also express fear and concern over the victim’s safety [31]. Among minoritized students, prior identity-driven violence can result in heightened fear of future victimization, depression, and anxiety on campus [32]. In a large sample of 1415 undergraduate students, Grinshteyn and colleagues found that students from various historically marginalized groups (i.e., Black, Hispanic, Middle Eastern, LGBTQ+) report a higher fear of discrimination and discriminatory violence compared to their majority-demographic counterparts [32]. Further, acts of targeted violence negatively impact students’ perceptions and expressions of campus climate and warmth and tarnish the institution’s reputation, which, in turn, influences the recruiting and retention of new students to the university over time [2]. Targeted violence directed at minoritized students has a direct impact on their academic and social wellbeing, as well as their perceptions of safety on campus.

Stressors unique to minoritized students (e.g., poor social/family support, internalized prejudicial attitudes, lack of structural support and resources, identity concealment) may contribute to significant deleterious outcomes following harassment [33,34]. For example, in a study of 934 LGBTQ+ youth, a structural equation model found that peer harassment victimization in school settings indirectly affected suicidal ideation and suicide attempts through school belongingness [33]. These stressors compound the existing challenges undergraduate students face in college settings and heighten the risk of experiencing psychological distress and academic difficulties. The perpetuation of cyber-related harassment is especially concerning as researchers highlight an association between online harassment and the later perpetration of offline harassment [23,27,35]. Given the increasing relevance of electronic behavior for college students’ experiences, additional research should clarify the prevalence of offline bias behavior and online bias behavior among college students.

According to Lazarus and Folkman’s Stress and Coping Theory, the level of distress experienced by victimized individuals is predicated on their cognitive appraisal of incidents and their coping responses to manage stressful experiences [36]. These behavioral responses are contextual in nature and impacted by an individual’s access to internal resources (e.g., adaptive coping skills) and external resources (e.g., confidential reporting avenues). Previous literature supports the hesitancy minoritized undergraduate students experience when deciding to formally engage law enforcement and formal agencies, yet it remains unclear how these students respond to instances of bias if not by involving external resources. As such, the current study aimed to contribute to the existing base of research by ascertaining the prevalence of identity-driven harassment among undergraduate students using an anonymous survey. This study also examined behavioral coping responses following instances of victimization and compared the experiences of minoritized (i.e., nonwhite, LGBTQ+) and majority-demographic students. Regarding bivariate comparisons, our hypotheses included that minoritized students would be more likely to experience identity-driven victimization and would be less likely to involve formal agencies following these incidents.

## 2. Method

A literature review and identification of appropriate measures began in the fall of 2019. Study procedures were developed throughout the spring of 2020, and the University Institutional Review Board (IRB) approved the study procedures, materials, and protocols before collecting data in April 2020. The current study falls under the “exempt” category and is due for re-review in 2025. Data for this study are derived from a cross-sectional electronic survey disseminated to undergraduate students via Qualtrics, an online survey platform. The relevant measures used in thus study are described below. Data collection began in the spring of 2020 and ended in May of 2023. Analyses were conducted using SPSS Version 28.

### 2.1. Participants

Participants included undergraduate students from a Midwestern university enrolled in psychology courses, and participants received three extra credit points for their courses for completing the survey. Students initially read a brief description of the study on an online university platform that advertises active studies in the psychology department seeking participants. Students interested in the study were directed to read a consent form and provide electronic consent to continue with the study. Participants must have been the age of majority in Nebraska (19 years of age) or older to participate. After completing the survey, participants were given an opportunity to read a debriefing form that describes the purpose of the study. Participants were informed of the anonymous nature of the study prior to providing consent. Four attention-check items were included throughout the survey to screen for valid responses.

### 2.2. Measures

Obsessive Relational Intrusion Short Form (ORI) [37]. A review of the literature revealed limited existing measures to examine identity-driven victimization and perpetration. Many existing measures were designed for children and adolescents who engage in antigay or racist bullying of other students, including name-calling and teasing. Thus, this study included a modified version of the Obsessive Relational Intrusion scale to broaden the range of behaviors assessed in the survey [37]. The ORI asks participants to provide frequency estimates for the prevalence of victimization and perpetration of harassment-related experiences. Participants rate behaviors on a scale from 0 (Never) to 4 (5 or more times). Behaviors range from making insults, to following a person, to engaging in physical or sexual assault. In the modified version of the ORI, participants indicate whether the behaviors were motivated by gender identity, sexuality, race, religion, nationality, political affiliation, or disability. Similar adaptations of existing victimization and perpetration measures to include identity-driven components have been made by other researchers [38]. Participants responded to modified versions of the ORI’s victimization and perpetration forms. The internal consistency for the ORI victimization version was α = 0.92 and 0.82 for the perpetration version.

Cyber-Obsessional Pursuit Scale (COP) [39]. Research indicates the growing rate of identity-driven harassment perpetrated via social media and computer technology [22]. To examine other cyber-related behaviors not captured in the ORI, participants also responded to the COP, which assesses online and cyber-related harassment experiences. The COP assesses a wide range of technology-specific behavior such as sending threatening text messages, hacking, and monitoring social media. Similar to the ORI, the COP was modified to collect data on whether these incidents were motivated by identity, and whether the individual perpetrated the behavior and/or was a victim of the behavior. In the current sample, the Cronbach’s alpha for the victimization version was α = 0.86 and 0.73 for the perpetration version.

Coping Responses Scale (CRS) [40]. The CRS is a 40-item measure that examines the frequency of behavioral responses following harassment and intrusive behavior as captured by the ORI and COP. Items are rated on a scale from 0 (Never) to 6 (Over 25 times) and include behaviors such as ignoring the issue, seeking therapy, relocating, involve law enforcement, and engage social support. Only participants who endorse victimization experiences completed the CRS. The internal consistency for the CRS was α = 0.95.

Campus Life Questionnaire (CLQ) [16]. The CLQ is a 15-item measure collecting information on bias victimization experiences that occur on campus grounds. A preamble is given prior to completing the form that reads, “Since you started at this university, have any of the following incidents happened to you on campus because of your (real or perceived) race/ethnicity, national origin, religion, sex, sexual orientation, physical or mental disability, or political orientation?” Items are rated as either “yes” or “no”. Sample items include “Been threatened with physical assault” and “Had verbal assaults directed at you”. For this study, a modified perpetration form of the CLQ was disseminated in tandem with the victimization form. The perpetration form was preceded with “Since you started at this university, have YOU done any of the following on-campus because of someone’s (real or perceived) race/ethnicity, national origin, religion, sex, sexual orientation, physical or mental disability, or political orientation?” Internal consistency analyses of the CLQ indicated a Cronbach’s alpha of 0.84 for the victimization version and 0.78 for the perpetration version.

## 3. Results

### 3.1. Sample Characteristics

A total of 3257 students aged 19 and older participated in the survey. Responses were screened for missing data and survey completion. Of the 3257 participants, 147 did not complete the survey and were excluded from the analyses. Of the remaining sample, 2000 (61.1%) provided valid and complete data, as indicated by satisfactory responses on all four attention checks included throughout the survey and completed survey data. This subset comprised the final sample of participants for this study. Table 1 illustrates the full demographic characteristics of the final sample. The majority of the sample identified as cisgender female (74.1%), white (82.1%), heterosexual (81.0%), and Christian (64.2%). The mean age of the sample was 20.11 (SD = 2.06; range = 19 to 45). Approximately half (50.1%) of participants identified as either very liberal or somewhat liberal, and the remaining majority identified as apolitical (21.2%) or somewhat conservative (22.2%). A minority of students identified as very conservative (6.2%).

### 3.2. Prevalence of Identity-Driven Harassment

Identity-driven victimization and perpetration were dichotomized to identify participants who endorsed any victimization and perpetration as indicated by nonzero responses on the ORI, COP, and CLQ. Table 2, Table 3 and Table 4 provide an overview of identity-driven experiences and identities targeted. A total of 1253 participants (62.6%) endorsed experiencing at least one instance of identity-driven harassment in their lifetime, with the most common form of bias being on the basis of sex (85.1%), followed by sexual orientation bias (40.3%) and racial bias (24.2%). Table 2, Table 3 and Table 4 illustrate the range of victimization behaviors reported on and off campus. The most common behaviors were receiving unwanted messages in-person (28.1%), experiencing an invasion of personal space (26.2%), experiencing verbal harassment on-campus (23.2%), and receiving harassing messages online (20.1%). Importantly, less severe instances of identity-driven victimization (i.e., verbal harassment) were more frequently endorsed compared to more severe forms of behavior (i.e., physical assault; see Table 2 and Table 3). Specifically, participants more often reported non-threatening, intrusive behavior such as being sent unwanted messages and having their personal space intruded upon (26.2% to 28.1%). A smaller percentage of participants endorsed experiencing surveillance and monitoring of their behavior and activities (11.5%). Threatening mediated contact (i.e., receiving threatening messages) was less common (1.3% to 9.8%), with the most frequent threatening message received being the perpetrator threatening self-harm. Physical approach behaviors such as in-person threats, assaultive behavior, and restraining the victim occurred in 1.4% to 7.3% of the sample. Victimization via unwanted sexual contact, sexual coercion, and verbal sexual harassment was reported by 14.4% to 19.7% of the sample. Of the sample, 5.3% reported being forced into sexual intercourse on-campus. Of those who indicated bias victimization, 45.5% reported harassment by an ex-partner, 42.5% by acquaintances, and 37.4% by friends. The most common response behaviors following bias victimization were ignoring the problem (76.6%), distancing from the person responsible (74.3%), minimizing the problem (73.1%), ignoring the person (57.9%), and engaging social support (57.6%).

The reporting of bias perpetration was comparatively less common, reflected in Table 5 and Table 6. A total of 277 participants (13.9%) reported engaging in bias-motivated perpetration behavior, with the most common motivations being sex-based (75.8%), sexual orientation-based (46.2%), and politically motivated (13.4%). The most common behaviors were sending harassing messages online (4.2%), expressing unwanted messages face-to-face (4.1%), sending tokens of affection online (3.9%), making exaggerated expressions of affection (3.2%), sending demanding messages online (3.2%), and watching others (2.9%). Of those who indicated bias perpetration, 59.6% reported harassing an ex-partner, 36.1% an acquaintance, and 18.4% a current partner.

Regarding identity-driven online harassment, students reported experiencing a wide range of behaviors including receiving unwanted, disclosive, and demanding messages (16.2% to 20.1%). Approximately 20% of the sample reported receiving unwanted sexually explicit pornographic material. Additionally, participants reported receiving online intimidation to expose their private information (7.4%) and sabotage their reputation (6.3%). A minority of the sample reported receiving threatening messages online (3.1%) or meeting an individual online or through social media and experiencing in-person harassment (0.3% to 1.4%).

Students from underrepresented backgrounds were more likely to report identity-driven harassment victimization than majority-demographic students (67.4% vs. 59.7%; *X*^2^(1) = 11.91; *p* = 0.001). Specifically, minoritized students were significantly more likely to report experiencing in-person bias harassment (49.9% vs. 41.3%; *X*^2^(1) = 14.14; *p* < 0.001), electronic bias harassment (40.9% vs. 32.9%; *X*^2^(1) = 13.28; *p* < 0.001) and on-campus bias harassment (43.1% vs. 37.6%; *X*^2^(1) = 6.01; *p* < 0.001). Subsequent analyses confirmed racial minority students were significantly more likely to experience race-based harassment (37.6% vs. 8.0%; *X*^2^(1) = 250.43; *p* < 0.001); sexual minority students were more likely to experience sexual orientation-based harassment (46.7% vs. 20.3%; *X*^2^(1) = 113.57; *p* < 0.001); transgender and gender non-conforming students were more likely to experience gender identity-based harassment compared to cisgender students (76.3% vs. 55.1%; *X*^2^(1) = 6.77; *p* = 0.01); and religious minority students were more likely to experience religious-based harassment (29.7% vs. 9.5%; *X*^2^(1) = 27.67; *p* < 0.001). Follow-up analyses indicated that minoritized students were at heightened risk of experiencing a wide range of behaviors within these modes of harassment, including receiving threatening messages, experiencing sexual harassment, and seeing offensive material on campus (see Table 7 for more details).

### 3.3. Behavioral Responses

Regardless of group membership, a large majority of students (76.6%) endorsed ignoring the problem as a response to harassment victimization. Nevertheless, minoritized students were significantly more likely to engage in various behaviors compared to majority-demographic students. Minoritized students who have experienced identity-driven harassment were significantly more likely to engage in informal action as opposed to formal action (58.8% vs. 24.4%; *X*^2^(1) = 31.00; *p* < 0.001); however, there were no significant differences between minoritized students and majority-demographic students regarding their likelihood to engage formal law enforcement, seek private assistance, or initiate formal action (e.g., file a complaint) following victimization (all *p*’s > 0.05).

The results revealed that minoritized students were significantly more likely to engage in an array of informal behaviors following bias victimization than majority-demographic students (see Table 8). Specifically, these students were more likely to engage in avoidance behaviors such as ignoring the person responsible and the problem and using strategies to minimize negative interactions with the individual (e.g., restricting electronic access, renegotiating relationships, and relocating). Additionally, they were more likely to engage in self-destructive behaviors (e.g., substance use, self-harm), seek therapy, and use self-reflection (all *p*’s < 0.05). There were no significant differences between minoritized and majority-demographic students regarding in-person bias (10.1% vs. 8.8%; *X*^2^(1) = 0.96; *p* = 0.34), electronic bias (9.3% vs. 8.1%; *X*^2^(1) = 0.84; *p* = 0.37), or on-campus harassment perpetration (3.2% vs. 2.2%; *X*^2^(1) = 2.02; *p* = 0.19).

## 4. Discussion

Identity-driven harassment and violence continues to persist in higher education institutions, disproportionately impacting historically marginalized and underrepresented students [17,41]. These acts can have severe consequences for victims, including significant psychological distress, social isolation, and reduced academic engagement [6,42,43]. This study explicitly responds to calls for increased attention to preventing identity-driven harassment and violence directed at students with marginalized identities by investigating the prevalence of in-person and electronic harassment victimization, perpetration, and problematic on-campus behavior [44,45]. This comprehensive approach to data collection provided a broader perspective on participants’ experiences.

It is essential to contextualize this dissertation by considering the broader geopolitical events that occurred from 2020 to 2023, which might have influenced participant responses. These events included George Floyd’s murder and the subsequent uprisings in the summer of 2020, the contentious 2020 Presidential election, the Supreme Court’s overturn of Roe v. Wade in the summer of 2022, and the ongoing COVID-19 pandemic, among others. Research has indicated that attitudes and behaviors can change in response to major social events [46]. It is possible minoritized students experienced more frequent and enduring instances of identity-driven harassment during this time of heightened geopolitical conflict that they otherwise may not have experienced. At the same time, it is possible victims of identity-driven harassment felt less comfortable sharing their experiences during this time period, resulting in underreporting. Further research endeavors are needed to replicate the prevalence of behaviors observed in this sample.

Data collection occurred over three full calendar years and initially involved over 3000 participants, ultimately resulting in a final sample of 2000 students. Sex- and gender-based harassment appeared more prevalent compared to other targeted identities. This finding might be attributed to most of the sample identifying as cisgender women, which inherently captured a substantial portion of sexist and misogynistic harassment. Notably, the finding that 85% of bias incidents had a sex-based component in our predominantly feminine sample aligns with the existing literature on college students who experience harassment on-campus—up to 68% of college women report that the harassment was sex-based, compared to 20% of men [19].

This study’s findings revealed that a significant portion of the sample, approximately 62.6% of students, reported experiencing identity-driven harassment victimization in their lifetime, with around 40% of these experiences occurring specifically on campus. These prevalence rates are consistent with those found in studies by DeKeseredy and colleagues [16]. While many participants acknowledged some form of bias and harassment, most of these experiences did not involve physical aggression or contact behavior. Most bias incidents reported by victims and perpetrators consisted of verbal offensive remarks, unwanted messages, and intrusive interactions. This finding aligns with the prior literature indicating that bias-motivated physical assaults on college campuses are relatively infrequent compared to other unwelcome behaviors [16]. Relatedly, studies have indicated that subtle forms of bias (e.g., microaggressions), are more common than overt forms of bias (e.g., slurs and epithets) [47] and our measures likely captured and encompassed both forms of behavior. Consistent with our hypothesis, underrepresented students were significantly more likely to experience bias-driven harassment targeting their identity within a marginalized group. This finding adds to the substantial body of evidence supporting the disproportionate targeting of minoritized students [16,17,41].

Notably, to the researcher’s knowledge, few empirical articles have directly compared the prevalence rates of bias and identity-driven harassment in university settings with those in the surrounding community. Researchers who have addressed this issue find that threatening behaviors are more common among college students, whereas actual instances of violence are more common in the community [48]. The elevated rates of bias incidents in this study may partially reflect an age effect. Rule-breaking and antisocial behavior tend to decrease as individuals transition into adulthood [49,50]; thus, using a collegiate sample may have contributed to the higher prevalence of identity-driven harassment, considering the inherent characteristics of undergraduate students, who are typically younger and more susceptible to impulsive and risky behavior.

One unique aspect of this study is the inclusion of measures to understand behavioral coping responses to bias victimization. Consistent with the previous literature, minoritized students were less likely to take formal action following identity-driven harassment [11,51]. Interestingly, majority-demographic students were similarly reluctant to report identity-driven incidents to formal authorities, which may suggest the hesitancy to make formal reports about this behavior stems from the nature of bias incidents as a whole. Traditionally underrepresented students were more inclined to respond to victimization by avoiding the issue and attempting to prevent further victimization without the assistance of others, for example, by relocating, restricting access, or directly addressing the perpetrator. These findings align well with a minority stress framework, where minoritized students who have experienced systemic discrimination and harassment over time may internalize a general distrust of others [52,53]. Moreover, evidence suggests that negative experiences with authorities in one domain (e.g., law enforcement) can “spill over” to affect trust in authorities in other domains (e.g., healthcare) [54], which might contribute to a broader negative perception of authorities who could assist on campus (i.e., housing, administration). Previous research also indicates that young minoritized students who report bias tend to perceive their situation as worse after reporting it [55], which could further influence their hesitancy to report. Students in this sample may have had similar negative experiences when making formal reports or harbored a general distrust of formal authorities due to past negative encounters, influencing their decision not to involve others following bias victimization. Unfortunately, the current study did not account for perceived satisfaction or effectiveness of various coping strategies, and this remains an area for future investigation.

Self-destructive behaviors, such as self-harm and substance use, emerged as more frequent responses among minoritized students than majority-demographic students. These findings reverberate results from various other studies, highlighting the prevalence of self-destructive behaviors among marginalized youth [56,57]. Drawing from a minority stress framework, the accumulation of minority-specific stressors (i.e., discrimination and bias) contributes to distress, suicidality, and harmful coping strategies. Researchers propose that internalized shame can result in inward expressions of negative emotions via self-harm and destructive behavior [58]. Paired with nonacceptance and structural powerlessness among historically marginalized students, it is possible that negative emotions (e.g., shame, self-blame) contributed to the elevated prevalence of harmful coping strategies. Indeed, meta-analyses suggest that the risk for suicidal ideation, suicide attempts, and alcohol and drug use is significantly higher among LGBTQ+ individuals [59,60] and Indigenous populations [61]. Among college students, suicidal thoughts and behaviors are more commonly expressed by women [62], racial-minority students [63], transgender students [64], and sexual-minority students [65]. Prior victimization experiences heighten the risk of mental health difficulties, self-harm, and suicidality among marginalized youth [42,43]. The results from this study expand upon the findings of other scholars by demonstrating that minoritized students who experience identity-driven harassment and bias are more likely to engage in harmful self-directed behavior as a direct response.

### Limitations and Future Directions

The current study presents with limitations that warrant mention. The modified survey instruments used in this study collected information about various identities targeted in victimization and perpetration behaviors (e.g., race, gender identity, disability status). Before disseminating this survey, no measures were available to the researchers’ knowledge that specifically examined identity-driven victimization and perpetration by addressing identity-related incidents. Consequently, modified versions of existing measures, such as the ORI, COP, and CLQ, were used instead. While internal consistencies for the modified measures were acceptable, the subtle modifications made to these measures may have influenced the instrument’s qualities. For example, the instruments used were not originally intended to collect information regarding targeted identities and without proper validation, it remains unclear whether our measures fully encapsulated participants’ experiences with bias and harassment. Thus, further construction and validation of tools to assess identity-driven experiences are needed. Additionally, the modified instruments measured the participant’s perception of their identity, not how others perceive them. Future studies could explore visibility, outness, and external perceptions to determine if these variables impact experiences like victimization and coping responses.

This study used a cross-sectional design in which participants provided data at a single time point in reference to previous actions that occurred retrospectively. This design limits the extent to which causal conclusions can be drawn from the data. The constructs measured in this study are dynamic and subject to change over time. The final sample consisted primarily of younger undergraduate students who were enrolled in introductory psychology courses, which may have contributed to a lower rate of students endorsing on-campus victimization and perpetration as they had comparatively less time on campus than more advanced students. A prospective, longitudinal, repeated-measures design that tracks students throughout their educational journey would provide a more comprehensive understanding of these experiences, enable more sophisticated statistical analyses, and enhance the quality of conclusions drawn. Such a design would also allow for comparisons between students in their first and final years of college to estimate the true prevalence of bias experiences throughout the college experience. Similarly, a majority of the current sample were white cisgender female students, which limits the generalizability of our findings. While we believe our study constitutes a meaningful step towards understanding undergraduate students’ experiences of bias, future research should aim to extend these findings in other diverse and representative samples.

As this study highlights, most incidents reported in this sample were primarily characterized as intrusive and unwanted interactions, with fewer severe incidents of interpersonal violence, such as physical assault. Nonetheless, certain concerning identity-driven behaviors were reported more frequently by participants reporting bias, including experiences such as sexual coercion and receiving pornographic images electronically. The existing literature indicates that experiences of sexual coercion and harassment are prevalent on college campuses, particularly among women and sexual minority students [66,67]. Future research should consider allocating resources to study the perpetration of these specific distressing behaviors, especially among college students. To extend these findings, researchers should also explore the identified risk factors in higher-risk samples, such as convicted hate crime offenders, and across various settings, such as workplaces and communities. This approach would help identify predictors of severe, hate-motivated violent behavior, going beyond the scope of harassment, and facilitate the generalization of findings to different contexts.

## 5. Practical Implications

Limitations notwithstanding, the findings from this study have relevant and novel implications for the management and prevention of concerning behavior among college students more broadly. The study revealed a high prevalence of identity-driven harassment on college campuses, particularly the targeting of historically marginalized groups based on sex, gender identity, and sexual orientation. The findings also shed light on how victims of such harassment often do not seek formal redress but instead engage in both positive actions, such as seeking therapy, and negative behaviors (e.g., substance use) to cope with distress. These acts of identity-driven violence are upsetting and stressful, and victims may feel compelled to manage these incidents independently without the help of available formal campus resources. Indeed, a study by McMahon and Seabrook found that minoritized students provided various reasons for not disclosing victimization, including concerns about burdening others and doubts about being believed if they reported the incidents [68]. University efforts should include resources dedicated to support marginalized students report incidents and engage with psychological help if needed.

The findings from this study align with those of previous studies that emphasize the need for culturally informed resources to encourage reporting among minoritized students [68,69,70]. Such efforts could involve providing specific training for officials responsible for disseminating resources and featuring diverse students in materials that promote these resources, making them more relatable to different groups. It is crucial to develop resources that are trauma-informed, inclusive, and sensitive to the hesitancy historically marginalized students often feel when considering reporting. Additionally, there should be ongoing measures to make reporting outlets more accessible to minoritized students, such as offering multiple reporting avenues, ensuring anonymity, and providing psychoeducation about their availability and utility. These enhancements would help identify and prevent problematic behavior on campus and potentially protect other historically marginalized students from bias victimization.

Colleges and universities have increasingly adopted threat assessment teams to prevent concerning behavior on campus. These multidisciplinary teams work together to identify warning signs and implement strategies to mitigate risk and reduce the likelihood of problematic behavior [71]. Although minimal research has explored the prevention of threatening bias-related behavior through a threat assessment framework, emerging evidence suggests that threat-assessment models could effectively address these incidents, as they often present with pre-incident warning signs like hostile looks or group-based threats [72]. Interestingly, post-hoc analyses suggested participants who reported engaging in threatening bias behavior also tended to engage in other concerning forms of behavior such as surveillance (58%) and nonthreatening behavior (86%). Significant differences emerged in the frequency of contacts of these behaviors as well; those who reported engaging in threatening bias behaviors engaged in surveillance as well as electronic and in-person non-threatening behavior on a more frequent basis than those who exclusively harassed via non-threatening means (all *p*’s < 0.001). Using multiple methods of contact and contacting a victim more frequently are both risk factors for physical-approach behavior, which is well established in the threat-assessment literature [73,74]. Thus, threat assessors may consider monitoring persons of concern for both frequency and diversity of contacts, as such behavior could represent an escalation of risk for violent behavior.

The findings of this study underscore the importance of improving the overall campus climate and enhancing perceptions of safety for minoritized students. Campus climate includes a student’s perception of the campus culture, the institution’s history and values, and the actions of administrative leaders [75]. Research has shown that victimized students who view the campus as less safe become more disengaged in their studies [20]. Moreover, specific factors related to the experiences of minoritized students, such as openness about identity, tend to enhance perceptions of the campus climate. Conversely, those who have experienced discrimination from instructors often have a more negative view of the campus climate [76]. In an extensive study of over 2000 undergraduate students, Coulter and Rankin found that students who perceive their campuses as more inclusive of sexual and gender minorities are less likely to report experiencing sexual assault on campus. The authors speculate that victims on inclusive campuses may feel less stigma in reporting concerning behavior, and bystanders may be more willing to intervene and prevent incidents from happening [77]. Thus, in addition to protecting victims, preventing identity-driven harassment on campus can improve campus climate and perceptions of safety, enhancing retention and the psychological well-being of historically marginalized students [78,79].

## 6. Conclusions

Identity-driven targeted violence and harassment are prevalent on college campuses nationwide, affecting students belonging to a wide range of diverse, underrepresented, and historically marginalized groups. Regardless of their background, every student deserves the opportunity to pursue higher education in a safe and supportive environment free from prejudice. Acts of bias on college campuses can hinder students’ academic progress, leading to self-destructive behaviors, isolation from their support networks, and a more negative perception of the university. These behaviors pose a considerable threat to the mental and physical wellbeing of students belonging to minoritized groups who already contend with multiple systemic barriers. The current study identified various identity-driven behaviors experienced among college students and the wide range of informal actions taken to ameliorate the impacts of these incidents independent of formal agency involvement. Researchers are encouraged to continue investigating the prevalence of bias on college campuses, their impact, and their underpinnings.

## Figures and Tables

**Table 1:** Demographic information of total sample (*N* = 2000).

Characteristic	*n*	% or Mean (SD)
Gender Identity		
Cisgender woman	1481	74.1
Cisgender man	481	24.1
Genderqueer/Non-binary	23	1.2
Transgender man	10	0.5
Transgender woman	2	0.1
Other gender identity	3	0.2
Race		
White/European American	1641	82.1
Hispanic/Latinx	222	11.1
Asian American/Pacific Islander	128	6.4
Black/African American	93	4.7
Biracial/Multiracial	60	3
Middle Eastern	32	1.6
Native American	22	1.1
Other racial identity	9	0.4
Sexual Orientation		
Heterosexual/Straight	1619	81
Bisexual	179	9
Unsure/Questioning	74	3.7
Gay	32	1.6
Pansexual	29	1.2
Lesbian	23	1.2
Queer	23	1.2
Asexual	16	0.8
Other sexual orientation	5	0.3
Religion		
Christian	1275	64.2
Agnostic	333	16.8
Atheist	182	9.2
Non-denominational	98	4.9
Muslim	30	1.5
Buddhist	22	1.1
Jewish	9	0.5
Hindu	4	0.2
Other religious identity	32	1.6
Political Orientation		
Very Liberal	394	19.7
Somewhat Liberal	608	30.4
Neither	423	21.2
Somewhat Conservative	448	22.4
Very Conservative	124	6.2
Age	2000	20.1 (2.1)
Socioeconomic Status		
High	95	4.8
Medium-High	612	30.6
Medium	811	40.6
Medium-Low	360	18
Low	121	6.1

Note. Total number of participants within each category may exceed the number of the total sample given the multiple category nature of the item. Other gender identities endorsed included agender, femme nonbinary, and genderfluid. Other racial identities endorsed included African, Armenian, Caribbean, East Indian, Hawaiian, Filipino, Portuguese, Sephardic, and Yazidi. Other sexual orientations included biromantic, demisexual, and heteroflexible. Other religions endorsed included Wicca, Pagan, Norse Pagan, Bahai, Free Spirit, and Spiritual/Secular.

**Table 2:** On-campus identity-driven victimization and perpetration.

Behavior	*n*	%	Behavior	*n*	%
On-campus identity-driven victimization	795	39.8	On-campus identity-driven perpetration		
Verbal harassment	464	23.2	Verbal harassment	32	1.6
Offensive phone calls/emails	123	6.2	Offensive phone calls/emails	14	0.7
Exposed to offensive online content	306	15.3	Exposing to offensive online content	7	0.4
Personal property damage	56	2.8	Personal property damage	2	0.1
Objects thrown	44	2.2	Objects thrown	1	0.1
Chased or followed	71	3.6	Chasing or following	0	0.0
Spat upon	22	1.1	Spitting upon	1	0.1
Threatened with physical assault	95	4.8	Threatened with physical assault	6	0.3
Been physically assaulted	72	3.6	Physically assaulting	3	0.2
Threatened with sexual behaviors	196	9.8	Threatened with sexual behaviors	1	0.1
Verbal sexual harassment	393	19.7	Verbal sexual harassment	2	0.1
Unwanted sexual touching	305	15.3	Unwanted sexual touching	2	0.1
Forced to have sexual intercourse	105	5.3	Forcing to have sexual intercourse	1	0.1
Threatened with a weapon	13	0.7	Threatened with a weapon	1	0.1
Attacked with a weapon	32	0.3	Attacked with a weapon	0	0.0

Note. Total number of participants within each category may exceed the number of the total sample given the multiple category nature of the item.

**Table 3:** Identity-driven victimization frequency by specific behavior.

Behavior	*n*	%	Behavior	*n*	%
In-person identity-driven victimization			Cyber identity-driven victimization		
Leaving unwanted gifts	258	12.9	Tokens of affection	270	13.5
Leaving unwanted messages	561	28.1	Messages of affection	401	20.1
Making exaggerated expressions	433	21.7	Disclosive messages	323	16.2
Following you	268	13.4	Demanding messages	363	18.2
Watching you	292	14.6	Pornographic messages	398	19.9
Intruding into interactions	288	14.4	Threatening messages	61	3.1
Invading personal space	523	26.2	Sexually harassing messages	233	11.7
Involving in unwanted activities	124	6.2	Threatening pictures	25	1.3
Invading personal property	103	5.1	Expose private info about you	147	7.4
Intruding upon friends/family	198	9.9	Pretending to be someone else	116	5.8
Monitoring behavior	229	11.5	Sabotaging private reputation	126	6.3
Approaching/surprising in public	165	8.3	Sabotaging professional rep	80	4.0
Obtaining private information	100	5.0	Attempting to disable computer	8	0.4
Invading property	46	2.3	Obtaining private info	33	1.7
Leaving threatening messages	109	5.5	Obtaining info about others	10	0.5
Physically restraining	146	7.3	Bugging car/home/office	1	0.1
Regulatory harassment	77	3.9	Altering electronic identity	12	0.6
Stealing/Damaging possessions	44	2.2	Taking over electronic identity	13	0.7
Threatening self-harm	196	9.8	Directing others to you	12	0.6
Threatening close others	62	3.1	Meeting online and following	28	1.4
Face-to-face threats	153	7.7	Meeting online and intruding	16	0.8
Threatening objects	25	1.3	Meeting online and threatening	18	0.9
Showing up in threatening ways	40	2.0	Meeting online and harming	6	0.3
Sexual coercion	287	14.4	Meeting online and stalking	24	1.2
Physical threats	74	3.7			
Physically hurting	107	5.4			
Kidnapping/Constraining	30	1.5			
Physical endangering	27	1.4			

Note. Total number of participants within each category may exceed the number of the total sample given the multiple category nature of the item.

**Table 4:** Identity-driven victimization by type of behavior experienced.

Behavior	*n*	%	Behavior	*n*	%
Any identity-driven victimization	1253	62.6	Cyber identity-driven victimization	720	36.0
Sex-based bias	1066	85.1	Sex-based bias	644	89.4
Gender identity bias	433	34.6	Gender identity bias	229	31.8
Racial bias	303	24.2	Racial bias	54	7.5
Sexuality bias	505	40.3	Sexuality bias	302	41.9
Religious bias	201	16.0	Religious bias	33	4.6
Political bias	275	21.9	Political bias	31	4.3
Nationality bias	87	6.9	Nationality bias	12	1.7
Disability bias	56	4.5	Disability bias	16	2.2
In-person identity-driven victimization	892	44.6	On-campus identity-driven victimization	798	39.9
Sex-based bias	768	86.1	Sex-based bias	591	74.3
Gender identity bias	278	31.2	Gender identity bias	237	29.8
Racial bias	112	12.6	Racial bias	226	28.4
Sexuality bias	359	40.2	Sexuality bias	209	26.3
Religious bias	93	10.4	Religious bias	126	15.8
Political bias	98	11.0	Political bias	203	25.5
Nationality bias	40	4.5	Nationality bias	49	6.2
Disability bias	19	2.1	Disability bias	37	4.7

Note. Total number of participants within each category may exceed the number of the total sample given the multiple category nature of the item.

**Table 5:** Identity-driven perpetration frequency by specific behavior.

Behavior	*n*	%	Behavior	*n*	%
In-person identity-driven perpetration	186	9.3	Cyber identity-driven perpetration	172	8.6
Leaving unwanted gifts	31	1.6	Tokens of affection	77	3.9
Leaving unwanted messages	82	4.1	Messages of affection	83	4.2
Making exaggerated expressions	68	3.4	Disclosive messages	39	2.0
Following them	14	0.7	Demanding messages	63	3.2
Watching them	59	2.9	Pornographic messages	43	2.2
Intruding into interactions	20	1.0	Threatening messages	4	0.2
Invading personal space	26	1.3	Sexually harassing messages	3	0.2
Involving in unwanted activities	5	0.3	Threatening pictures	0	0.0
Invading personal property	3	0.2	Expose private info about them	9	0.5
Intruding upon friends/family	12	0.6	Pretending to be someone else	25	1.3
Monitoring behavior	41	2.1	Sabotaging private reputation	5	0.3
Approaching/surprising in public	13	0.7	Sabotaging professional rep	5	0.3
Obtaining private information	14	0.7	Attempting to disable computer	1	0.1
Invading property	1	0.1	Obtaining private info	7	0.4
Leaving threatening messages	2	0.1	Obtaining info about others	3	0.2
Physically restraining	4	0.2	Bugging car/home/office	0	0.0
Regulatory harassment	0	0.0	Altering electronic identity	1	0.1
Stealing/Damaging possessions	2	0.1	Taking over electronic identity	1	0.1
Threatening self-harm	11	0.5	Directing others to them	0	0.0
Threatening close others	2	0.1	Meeting online and following	1	0.1
Face-to-face threats	8	0.4	Meeting online and intruding	1	0.1
Threatening objects	0	0.0	Meeting online and threatening	0	0.0
Showing up in threatening ways	0	0.0	Meeting online and harming	0	0.0
Sexual coercion	6	0.3	Meeting online and stalking	0	0.0
Physical threats	1	0.1			
Physically hurting	7	0.4			
Kidnapping/Constraining	2	0.1			
Physical endangering	0	0.0			

**Table 6:** Identity-driven perpetration by targeted identity and type of behavior perpetrated.

Behavior	*n*	%	Behavior	*n*	%
Any identity-driven perpetration	277	13.9	Cyber identity-driven perpetration	172	8.6
Sex-based bias	210	75.8	Sex-based bias	144	83.7
Gender identity bias	81	29.2	Gender identity bias	53	30.8
Racial bias	30	10.8	Racial bias	9	5.2
Sexuality bias	128	46.2	Sexuality bias	86	50.0
Religious bias	23	8.3	Religious bias	5	2.9
Political bias	37	13.4	Political bias	9	5.2
Nationality bias	13	4.7	Nationality bias	4	2.3
Disability bias	13	4.7	Disability bias	3	1.7
In-person identity-driven perpetration	186	9.3	On-campus identity-driven perpetration	52	2.6
Sex-based bias	142	76.3	Sex-based bias	16	30.8
Gender identity bias	60	32.3	Gender bias	10	19.2
Racial bias	13	7.0	Racial bias	14	26.9
Sexuality bias	94	50.5	Sexuality bias	8	15.4
Religious bias	13	7.0	Religious bias	9	17.3
Political bias	14	7.5	Political bias	24	46.2
Nationality bias	4	2.2	Nationality bias	4	7.7
Disability bias	9	4.8	Disability bias	1	1.9

Note. Total number of participants within each category may exceed the number of the total sample given the multiple category nature of the item.

**Table 7:** Significant differences in identity-driven victimization between minority and majority demographic students.

	Minority Students		Majority Students		Chi-Square	*p*
	(*n* = 772)		(*n* = 1228)			
	*n*	%	*n*	%		
In-person victimization behavior						
Receiving unwanted messages	243	31.5	318	25.9	7.32	0.008
Being watched	141	18.3	151	12.3	13.54	<0.001
Intruding uninvited	146	18.9	142	11.6	20.76	<0.001
Involving in unwanted ways	65	8.4	59	4.8	10.65	0.002
Intruding on family/friends	103	13.3	95	7.7	16.7	<0.001
Obtaining private information	55	7.1	45	3.7	11.95	0.001
Receiving threatening messages	62	8	47	3.8	16.26	<0.001
Being physically restrained	83	10.8	63	5.1	22.13	<0.001
Receiving self-harm threats	93	12	103	8.4	7.18	0.009
Receiving verbal threats	89	11.5	64	5.2	26.77	<0.001
Experiencing sexual coercion	138	17.9	149	12.1	12.72	<0.001
Receiving physical threats	41	5.3	33	2.7	9.16	0.003
Being physically hurt	64	8.3	43	3.5	21.46	<0.001
Being kidnapped/constrained	20	2.6	10	0.8	10.12	0.002
Being physically endangered	18	2.3	9	0.7	9.1	0.004
Electronic victimization behavior						
Receiving electronic tokens of affection	127	16.5	143	11.6	9.38	0.002
Receiving excessively disclosive messages	152	19.7	171	13.9	11.63	0.001
Being sent pornographic messages	181	23.4	217	17.7	9.92	0.002
Receiving threatening electronic messages	39	5.1	22	1.8	17.04	<0.001
Receiving sexually harassing messages	117	15.2	116	9.4	15.01	<0.001
Having private info exposed online	72	9.3	75	6.1	7.21	0.008
Being catfished	59	7.6	57	4.6	7.81	0.006
Having reputation sabotaged	63	8.2	63	5.1	7.37	0.008
Obtaining private information	20	2.6	13	1.1	6.86	0.01
Altering electronic data	9	1.2	3	0.2	6.75	0.01
On-campus victimization						
Experiencing verbal harassment	202	26.2	262	21.3	6.21	0.01
Being exposed to offensive material	164	21.2	142	11.6	34.27	0

Note. Significance set at *p* ≤ 0.01 to avoid likelihood of committing a Type I error due to multiple comparisons.

**Table 8:** Significant differences in behavior following identity-driven victimization.

Coping Response Behavior	Minority Students (*n* = 485)		Majority Students (*n* = 667)		Chi-Square	*p*
	*n*	%	*n*	%		
Ignore the problem *(e.g., wait, assume problem will go away on its own, etc.)*	392	80.3	494	74.0	6.40	0.01
Seek therapy *(e.g., invest time and effort into hobbies, drugs, exercise, medicine, therapeutic activities such as a massage, meditation, exercise, watching television, internet, etc.)*	157	32.4	153	22.9	12.70	<0.001
Seek meaning in general *(e.g., invest time and effort into making sense of your situation, trying to find a reason, etc.)*	261	53.8	290	43.5	12.02	0.001
Seek meaning in context *(e.g., invest time and effort into religion, philosophy, education, literature, etc.)*	179	36.9	208	31.2	4.12	0.04
Engage in self-destruction *(e.g., using drugs or alcohol, doing addictive things, attempting suicide, etc.)*	151	31.1	135	20.2	17.86	<0.001
Attempt to deter future behavior *(e.g., carry air horn or mace, show weapon, seek self-defense training, put security stickers on car and home windows, etc.)*	173	35.7	180	27.0	9.86	0.002
Behave cautiously *(e.g., make plans of action and escape, become more aware of environment, become more conservative or careful in daily routing, etc.)*	237	48.9	283	42.5	4.60	0.04
Ignore the person *(e.g., avoid eye contact, be non-responsive to pursuer’s talk and behaviors)*	303	62.5	364	54.6	7.19	0.008
Control future interactions *(e.g., avoid asking questions, use closed body orientation, stand/sit closer with others during conversation, etc.)*	240	49.5	284	42.6	5.40	0.02
Restrict electronic access (e.g., caller ID, *69, change email address, contact ISP or internet provider to block certain contact sources, etc.)	172	35.5	190	28.5	6.35	0.01
Relocate *(e.g., change jobs, change address, change classes, change hobby/recreational locations, etc.)*	58	12.0	47	7.0	8.18	0.005
Renegotiate relationship *(e.g., discuss pursuer’s own preferred relationship objectives to arrive at mutual definition: just be friends, just be colleagues, reconciliation of previous relationship, etc.)*	158	32.6	180	27.0	4.32	0.04
Use verbal aggression *(e.g., yell at, criticize, insult, make fun of, show anger, annoyance, frustration, use harsh or hostile voice, write strongly worded email, etc.)*	119	24.5	111	16.6	10.95	0.001

Note. Total number of participants within each category may exceed the number of the total sample given the multiple category nature of the item.

## Data Availability

Inquiries regarding the availability of the data included in this study may be directed to the corresponding author.

## References

[1] Bender A.K., Lauritsen J.L. (2021). Violent Victimization among Lesbian, Gay, and Bisexual Populations in the United States: Findings from the National Crime Victimization Survey, 2017–2018. Am. J. Public Health.

[2] Regehr C., Glancy G.D., Carter A., Ramshaw L. (2017). A Comprehensive Approach to Managing Threats of Violence on a University or College Campus. Int. J. Law Psychiatry.

[3] Bliuc A.M., Faulkner N., Jakubowicz A., McGarty C. (2018). Online Networks of Racial Hate: A Systematic Review of 10 years of Research on Cyber-Racism. Comput. Hum. Behav..

[4] Poteat V.P., Rivers I., Vecho O., Poteat V.P., Rivers I., Vecho O. (2015). The Role of Peers in Predicting Students’ Homophobic Behavior: Effects of Peer Aggression, Prejudice, and Sexual Orientation Identity Importance. Sch. Psych. Rev..

[5] Rankin S.R. (2005). Campus Climates for Sexual Minorities. New Dir. Stud. Serv..

[6] Cramer R.J., Wright S., Long M.M., Kapusta N.D., Nobles M.R., Gemberling T.M., Wechsler H.J. (2018). On Hate Crime Victimization: Rates, Types, and Links with Suicide Risk among Sexual Orientation Minority Special Interest Group Members. J. Trauma Dissociation.

[7] Sibley C.G., Duckitt J. (2008). Personality and Prejudice: A Meta-Analysis and Theoretical Review. Personal. Soc. Psychol. Rev..

[8] Parrott D.J. (2008). A Theoretical Framework for Antigay Aggression: Review of Established and Hypothesized Effects within the Context of the General Aggression Model. Clin. Psychol. Rev..

[9] United States Federal Bureau of Investigation (2019). Uniform Crime Reporting Handbook: UCR.

[10] Han S., Riddell J.R., Piquero A.R. (2023). Anti-Asian American Hate Crimes Spike During the Early Stages of the COVID-19 Pandemic. J. Interpers. Violence.

[11] Pezzella F.S., Fetzer M.D., Keller T. (2019). The Dark Figure of Hate Crime Underreporting. Am. Behav. Sci..

[12] Vergani M., Navarro C. (2021). Hate Crime Reporting: The Relationship between Types of Barriers and Perceived Severity. Eur. J. Crim. Policy Res..

[13] Heilbrun K., Dvoskin J., Heilbrun A. (2009). Toward Preventing Future Tragedies: Mass Millings on College Campuses, Public Health, and Threat/Risk Assessment. Psychol. Inj. Law.

[14] Scalora M., Simons A., VanSlyke S. (2010). Campus Safety: Assessing and Managing Threats. FBI Law Enforc. Bull..

[15] Brady P.Q., Nobles M.R., Bouffard L.A. (2017). Are College Students Really at a Higher Risk for Stalking? Exploring the Generalizability of Student Samples in Victimization Research. J. Crim. Justice.

[16] DeKeseredy W., Nolan J.J., Hall-Sanchez A. (2019). Hate Crimes and Bias Incidents in the Ivory Tower: Results from a Large-Scale Campus Survey. Am. Behav. Sci..

[17] Rankin S.R. (2003). Campus Climate for Gay, Lesbian, Bisexual and Transgender People: A National Perspective.

[18] Rankin S.R., Reason R.D. (2005). Differing Perceptions: How Students of Color and White Students Perceive Campus Climate for Underrepresented Groups. J. Coll. Stud. Dev..

[19] Reason R.D., Rankin S.R. (2006). Perception of Harassment on Campus. Coll. Stud. Aff. J..

[20] Kammer-Kerwick M., Wang A., McClain T., Hoefer S., Swartout K.M., Backes B., Busch-Armendariz N. (2021). Sexual Violence among Gender and Sexual Minority College Students: The Risk and Extent of Victimization and Related Health and Educational Outcomes. J. Interpers. Violence.

[21] Ortega-Williams A., Booth J., Fussell-Ware D., Lawrence Y., Pearl D., Chapman N., Allen W., Reid-Moore A., Overby Z. (2021). Using Ecological Momentary Assessments to Understand Black Youths’ Experiences of Racism, Stres, and Safety. J. Res. Adolesc..

[22] Henry J.S. (2013). Bias-Based Cyberbullying: The next Hate Crime Frontier?. Crim. Law Bull..

[23] Watts L.K., Wagner J., Velasquez B., Behrens P.I. (2017). Cyberbullying in Higher Education: A Literature Review. Comput. Hum. Behav..

[24] Zalaquett C.P., Chatters S.J. (2014). Cyberbullying in College: Frequency, Characteristics, and Practical Implications. SAGE Open.

[25] Gleason N.W. (2018). Higher Education in the Era of the Fourth Industrial Revolution.

[26] Xu M., David J.M., Kim S.H. (2018). The Fourth Industrial Revolution: Opportunities and Challenges. Int. J. Financ. Res..

[27] Hinduja S., Patchin J.W. (2008). Cyberbullying: An Exploratory Analysis of Factors Related to Offending and Victimization. Deviant Behav..

[28] Saha K., Chandrasekharan E., De Choudhury M. Prevalence and Psychological Effects of Hateful Speech in Online College Communities. Proceedings of the 11th ACM Conference on Web Science.

[29] Schenk A.M., Fremouw W.J., Keelan C.M. (2013). Characteristics of College Cyberbullies. Comput. Hum. Behav..

[30] Mengo C., Black B.M. (2016). Violence Victimization on a College Campus: Impact on GPA and School Dropout. J. Coll. Stud. Retent. Res. Theory Pract..

[31] Pemberton A., Vanfraechem I. (2015). Victims’ Victimization Experiences and Their Need for Justice. Victims and Restorative Justice.

[32] Grinshteyn E., Whaley R., Couture M.C. (2022). High Fear of Discriminatory Violence among Racial, Gender, and Sexual Minority College Students and Its Association with Anxiety and Depression. Int. J. Environ. Res. Public Health.

[33] Hatchel T., Merrin G.J., Espelage D. (2019). Peer Victimization and Suicidality among LGBTQ Youth: The Roles of School Belonging, Self-Compassion, and Parental Support. J. LGBT Youth.

[34] Testa R.J., Michaels M.S., Bliss W., Rogers M.L., Balsam K.F., Joiner T. (2017). Suicidal Ideation in Transgender People: Gender Minority Stress and Interpersonal Theory Factors. J. Abnorm. Psychol..

[35] Williams M.L., Burnap P., Javed A., Liu H., Ozalp S. (2020). Hate in the Machine: Anti-Black and Anti-Muslim Social Media Posts as Predictors of Offline Racially and Religiously Aggravated Crime. Br. J. Criminol..

[36] Lazarus R.S., Folkman S. (1987). Transactional Theory and Research on Emotions and Coping. Eur. J. Pers..

[37] Cupach W.R., Spitzberg B.H. (2004). Obsessive Relational Intrusion and Stalking. The Dark Side of Close Relationships.

[38] Nappa M.R., Morelli M., Bianchi D., Baiocco R., Cattelino E., Chirumbolo A. (2019). The Dark Side of Homophobic Bullying: The Moderating Role of Dark Triad Traits in the Relationship between Victim and Perpetrator. Rass. Psicol..

[39] Spitzberg B.H. (2002). Cyberstalking and the Technologies of Interpersonal Terrorism. New Media Soc..

[40] Spitzberg B.H., Cupach W.R. (2001). Paradoxes of Pursuit: Toward a Relational Model of Stalking-Related Phenomena. Stalking Crimes and Victim Protection: Prevention, Intervention, Threat Assessment, and Case Management.

[41] Myers W., Turanovic J.J., Lloyd K., Pratt T. (2020). The Victimization of LGBTQ Students at School: A Meta-Analysis. J. Sch. Violence.

[42] Blosnich J.R., Bossarte R. (2013). Drivers of Disparity: Differences in Sociall-Based Risk Factors of Self-Injurious and Suicidal Behaviors among Sexual Minority College Students. J. Am. Coll. Health.

[43] Williams, Jones C., Arcelus J., Townsend E., Lazaridou A., Michail M. (2021). A Systematic Review and Meta-Analysis of Victimisation and Mental Health Prevalence among LGBTQ + Young People with Experiences of Self-Harm and Suicide. PLoS ONE.

[44] Chakraborti N., Hardy S.J. (2017). Beyond Empty Promises? A Reality Check for Hate Crime Scholarship and Policy. Safer Communities.

[45] Gauthier J., Medina K., Dierkhising C. (2021). Analysis of Hate Crimes in Transgender Communities. J. Hate Stud..

[46] Darling-Hammond S., Michaels E.K., Allen A.M., Chae D.H., Thomas M.D., Nguyen T.T., Mujahid M.M., Johnson R.C. (2020). After “The China Virus” Went Viral: Racially Charged Coronavirus Coverage and Trends in Bias against Asian Americans. Health Educ. Behav..

[47] Boysen G.A., Vogel D.L., Cope M.A. (2009). Incidents of Bias in College Classrooms: Instructor and Student Perceptions. J. Divers. High. Educ..

[48] Leonard K.E., Quigley B.M., Collins R.L. (2002). Physical Aggression in the Lives of Young Adults: Prevalence, Location, and Severity. J. Interpers. Violence.

[49] Jennings W.G., Reingle J.M. (2012). Reingle On the Number and Shape of Developmental/Life-Course Violence, Aggression, and Delinquency Trajectories: A State-of-the-Art Review. J. Crim. Justice.

[50] Piquero A.R., Jennings W.G., Barnes J.C. (2012). Violence in Criminal Careers: A Review of the Literature from a Developmental Life-Course Perspective. Aggress. Violent Behav..

[51] (2014). Giannasi Policing and Hate Crime. The Routledge International Handbook on Hate Crime.

[52] Meyer I.H. (1995). Minority Stress and Mental Health in Gay Men. J. Health Soc. Behav..

[53] Meyer I.H. (2003). Prejudice, Social Stress, and Mental Health in Lesbian, Gay, and Bisexual Populations: Conceptual Issues and Research Evidence. Psychol. Bull..

[54] Feelemyer J., Duncan D.T., Remch M., Kaufman J.S., Cleland C., Geller A.B., Dyer T.V., Scheidell J.D., Turpin R., Brewer R. (2023). Associations between Police Harassment and Distrust in and Reduced Access to Healthcare among Black Sexual Minority Men: A Longitudinal Analysis of HPTN 061. PLoS ONE.

[55] Mendez J.J., Bauman S., Sulkowski M.L., Davis S., Nixon C. (2016). Racially-Focused Peer Victimization: Prevalence, Psychosocial Impacts, and the Influence of Coping Strategies. Psychol. Violence.

[56] Drescher C.F., Kassing F., Mahajan A., Lara M. (2023). The Impact of Transgender Minority Stress and Emotion Regulation on Suicidality and Self-Harm. Psychol. Sex..

[57] Kidd G., Marston L., Nazareth I., Osborn D., Pitman A. (2023). Suicidal Thoughts, Suicide Attempt and Non-Suicidal Self-Harm amongst Lesbian, Gay and Bisexual Adults Compared with Heterosexual Adults: Analysis of Data from Two Nationally Representative English Household Surveys. Soc. Psychiatry Psychiatr. Epidemiol..

[58] Tangney J., Stuewig J., Mashek D. (2007). Moral Emotions and Moral Behaviior. Annu. Rev. Psychol..

[59] King M., Semlyen J., Tai S.S., Killaspy H., Osborn D., Popelyuk D., Nazareth I. (2008). A Systematic Review of Mental Disorder, Suicide, and Deliberate Self Harm in Lesbian, Gay and Bisexual People. BMC Psychiatry.

[60] Marchi M., Arcolin E., Fiore G., Travascio A., Amaddeo F., Converti M., Fiorillo A., Pinna F., Ventriglio A., Galeazzi G.M. (2022). Self-Harm and Suicidality among LGBTIQ People: A Systematic Review and Meta-Analysis. Int. Rev. Psychiatry.

[61] Troya M.I., Spittal M.J., Pendrous R., Crowley G., Gorton H.C., Russell K., Byrne S. (2022). Suicide Rates amongst Individuals from Ethnic Minority Backgrounds: A Systematic Review and Meta-Analysis. eClinicalMedicine.

[62] Mortier P., Cuijpers P., Kiekens G., Auerbach R.P., Demyttenaere K., Green J.G. (2018). The Prevalence of Suicidal Thoughts and Behaviours among College Students: A Meta-Analysis. Psychol. Med..

[63] Sa C., Cho C. (2020). Sex and Racial/Ethnic Differences in Suicidal Consideration and Suicide Attempts among US College Students. Am. J. Health Behav..

[64] Effrig J.C., Bieschke K.J., Locke B.D. (2011). Examining Victimization and Psychological Distress in Transgender College Students. J. Coll. Couns..

[65] Mortier P., Auerbach R., Alonso J., Bantjes J., Benjet C., Cuijpers P., Ebert D., Greif Green J., Hasking P., Nock M. (2018). Suicidal Thoughts and Behaviors among First-Year College Students—Results from the WMH-ICS Project. Physiol. Behav..

[66] Klein L.B., Martin S. (2021). Sexual Harassment of College and University Students: A Systematic Review. Trauma Violence Abus..

[67] Steele B., Esposti M.D., Mandeville P., Hamnett G., Nye E., Humphreys D.K. (2021). Sexual Violence among Students Attending a Higher Education Institution in the UK (OUR SPACE): A Across-Sectional Survey. Lancet.

[68] McMahon S., Seabrook R.C. (2020). Reasons for Nondisclosure of Campus Sexual Violence by Sexual and Racial/Ethnic Minority Women. J. Stud. Aff. Res. Pract..

[69] Edwards K.M., Sylaska K.M., Barry J.E., Moynihan M.M., Banyard L., Cohn E.S., Walsh W.A., Ward S.K. (2015). Physical Dating Violence, Sexual Violence, and Unwanted Pursuit Victimization: A Comparison of Incidence Rates among Sexual-Minority and Heterosexual College Students. J. Interpers. Violence.

[70] Ollen E.W., Ameral V.E., Reed K.P., Hines D.A. (2017). Sexual Minority College Students’ Perceptions on Dating Violence and Sexual Assault. J. Couns. Psychol..

[71] Deisinger E.R.D., Scalora M.J. (2016). Threat Assessment and Management in Higher Education in the United States: A Review of the 10 Years since the Mass Casualty Incident at Virginia Tech. J. Threat Assess. Manag..

[72] Siddoway K. (2021). Targeted Violence on Campus: A Comparison of Exposure and Response to Bias and Otherwise Motivated Potential Pre-Incident Behavior on a College Campus. Ph.D. Thesis.

[73] Meloy, Hoffmann J., Guldimann A., James D. (2012). The Role of Warning Behaviors in Threat Assessment: An Exploration and Suggested Typology. Behav. Sci. Law.

[74] Scalora M.J., Baumgartner J., Zimmerman W., Callaway D., Hatch Maillette M., Covell C., Palarea R., Krebs J., Washington D. (2002). An Epidemiological Assessment of Problematic Contacts to Members of Congress. J. Forensic Sci..

[75] Hart J., Fellabaum J. (2008). Analyzing Campus Climate Studies: Seeking to Define and Understand. J. Divers. High. Educ..

[76] Tetreault P., Fette R., Meidlinger P., Hope D. (2013). Perceptions of Campus Climate by Sexual Minorities. J. Homosex..

[77] Coulter R., Rankin S. (2018). College Sexual Assault and Campus Climate for Sexual- and Gender- Minority Undergraduate Students. J. Interpers. Violence.

[78] Koo K.K., Baker I., Yoon J. (2021). The First Year of Acculturation: A Longitudinal Study on Acculturative Stress and Adjustment among First-Year International College Students. J. Int. Stud..

[79] Strayhorn T.L. (2013). Measuring Race and Gender Differences in Undergraduate Students’ Perceptions of Campus Climate and Intentions to Leave College: An Analysis in Black and White. J. Stud. Aff. Res. Pract..

